# Perioperative intravenous iron to treat patients with fractured hip surgery: A systematic review and meta‐analysis

**DOI:** 10.1002/hsr2.633

**Published:** 2022-05-22

**Authors:** Rhona C. F. Sinclair, Miranda J. A. Bowman, Iain K. Moppett, Michael A. Gillies

**Affiliations:** ^1^ Consultant Anaesthetist Royal Victoria Infirmary Newcastle upon Tyne UK; ^2^ The Medical School University of Newcastle Newcastle upon Tyne UK; ^3^ Professor of Anaesthesia and Perioperative Medicine, Anaesthesia and Critical Care Research Group, Division of Clinical Neuroscience, Queens Medical Centre University of Nottingham Nottingham UK; ^4^ Consultant and Honorary Professor in Intensive Care Royal Infirmary of Edinburgh, NHS Lothian Edinburgh UK

**Keywords:** anemia, hip fracture, intravenous iron

## Abstract

**Background:**

Treatment of preoperative anemia with intravenous iron is common within elective surgical care pathways. It is plausible that this treatment may improve care for people with hip fractures many of whom are anemic because of pre‐existing conditions, fractures, and surgery.

**Objective:**

To review the evidence for intravenous iron administration on outcomes after hip fracture.

**Design:**

We followed a predefined protocol and conducted a systematic review and meta‐analysis of the use of intravenous iron to treat anemia before and after emergency hip fracture surgery. The planned primary outcome was a difference in length of stay between those treated with intravenous iron and the control group. Other outcomes analyzed were 30‐day mortality, requirement for blood transfusion, changes in quality of life, and hemoglobin concentration on discharge from the hospital.

**Data Sources:**

EMBASE, MEDLINE, The Cochrane Library (CENTRAL, DARE) databases, Clinicaltrials.gov, and ISRCTN trial registries. Date of final search March 2022.

**Eligibility Criteria:**

Adult patients undergoing urgent surgery for hip fracture. Studies considered patients who received intravenous iron and were compared with a control group.

**Results:**

Four randomized controlled trials (RCT, 732 patients) and nine cohort studies (2986 patients) were included. The RCTs were at low risk of bias, and the nonrandomized studies were at moderate risk of bias. After metanalysis of the RCTs there was no significant difference in the primary outcome, length of hospital stay, between the control group and patients receiving intravenous iron (mean difference: −0.59, 95% confidence interval [CI]; −1.20 to 0.03; *I* ^2^ = 30%, *p* = 0.23). Intravenous iron was not associated with a difference in 30‐day mortality (*n* = 732, OR: 1.14, 95% CI: 0.62−2.1; *I* ^2^ = 0%, *p* = 0.50), nor with the requirement for transfusion (*n* = 732, OR: 0.85, 95% CI: 0.63−1.14; *I* ^2^ = 0%, *p* < 0.01) in the analyzed RCTs. Functional outcomes and quality of life were variably reported in three studies.

**Conclusion:**

The evidence on the use of intravenous iron in patients with hip fracture is low quality and shows no difference in length of acute hospital stay and transfusion requirements in this population. Improved large, multicentre, high‐quality studies with patient‐centered outcomes will be required to evaluate the clinical and cost‐effectiveness of this treatment.

## 1 INTRODUCTION

Fragility hip fracture affects over 70,000 patients each year in the United Kingdom^1^ and over 250,000 annually in the United States.^2^ Anemia is a common finding in these patients. In a recent report, 44% were anemic on admission to hospital rising to 87% after surgery.^3^ Systematic reviews suggest that anemia in this population is associated with increased mortality^4^ and perioperative blood transfusion is common (approximately 30% of patients).

Patient blood management strategies (PBM) are currently recommended before surgery by NHS Blood and Transplant, The National Institute for Health and Care Excellence, and expert groups.^5^, ^6^, ^7^, ^8^ PBM has been incorporated into the pathways of care for older patients undergoing elective orthopedic surgery, leading to a reduction in the requirement for perioperative blood transfusion.^3^, ^7^ Intravenous iron preparations are now regularly used ahead of elective surgeries that are associated with significant blood loss. Commonly used preparations include ferric carboxymaltose up to 1000 mg or iron isomaltoside up to 20 mg/kg given intravenously as infusions over 15–30 min.

Observational studies link perioperative anemia with poor postoperative outcomes, increased mortality, and a reduction in quality of life following surgery for hip fracture^4^ and other types of surgery.^9^, ^10^ Uncorrected anemia after hip fracture may impede functional recovery in this group of patients^11^, ^12^ and may have a lasting effect on recovery and impact on quality of life.^13^ Other trials have assessed the use of interventions such as IV iron to minimize anemia and exposure to blood products in other surgical groups.^14^, ^15^ The use of IV iron is not currently routinely employed after hip fracture in the United Kingdom.^16^ Potentially, the use of intravenous iron is an attractive option to improve outcomes for the hip fracture population, by avoiding exposure to blood products and improving the quality of recovery.

We undertook a systematic review to assess the evidence and effect of intravenous iron preparations on the duration of acute hospitalization, mortality, blood transfusion, quality of life, and discharge hemoglobin in patients presenting to surgery with hip fracture.

## 2 METHOD

This systematic review was conducted in accordance with our predefined protocol registered prospectively on PROSPERO (CRD42020171197), and the preferred reporting items for systematic reviews and meta‐analyses (PRISMA).^17^

### 2.1 Search strategy and study inclusion criteria

We searched EMBASE, MEDLINE, The Cochrane Library (CENTRAL, DARE) databases, Clinicaltrials.gov, and ISRCTN trial registries between January 2000 and January 2021. The search was repeated on 31st March 2022 to check for additional studies and one additional study met the inclusion criteria and was added to the meta‐analysis. Studies eligible for inclusion were randomized or observational trials of adult patients (age over 18 years) undergoing emergency or urgent surgery for hip fracture and who received intravenous iron (any preparation) in the perioperative period. All study designs were considered if intravenous iron was compared with a control group of patients who did not receive this intervention and where one of the outcomes of interest was reported. Studies published in any language were considered. A full description of the strategy used is contained in Supporting Information S1.

Two review authors (R. C. F. Sinclair and M. J. A. Bowman) independently screened citations from the systematic search and extracted data. Discrepancies or disagreements were then adjudicated by a further author M. A. Gillies). Manual searching was also used to identify other reports, and all references in the selected full‐text articles were reviewed to identify further possible studies for inclusion. We excluded studies where any of the outcomes of interest were not reported, where patients undergoing elective hip surgery were studied, and where there was no comparison group (who did not receive the intervention) reported.

The primary outcome for this review was the effect of intravenous iron after hip fracture surgery on the duration of acute hospitalization after emergency surgery (length of stay, measured in days). Secondary outcomes were mortality at 30 days/discharge and 90 days following surgery, change in the quality of life after emergency hip surgery, and postoperative red cell transfusion and hemoglobin concentration at acute hospital discharge.

### 2.2 Data analysis

Statistical analysis and data presentation were performed using R (R Foundation, Vienna)^18^ and the meta‐package.^19^ For randomized trials risk of bias was assessed using the Cochrane Collaboration tool for randomized controlled trials.^20^ The Newcastle Ottawa scale was used for observational studies.^21^ Funnel plots were used to detect publication bias for primary or secondary endpoints.

Statistical heterogeneity between studies was estimated using *Χ* ^2^ and *I* ^2^ tests. A *p*‐value of 0.1 was used to denote the statistical significance of heterogeneity. For *I* ^2^ tests, cut‐offs of 25%, 50%, and 75% were used to demonstrate the presence of low, moderate, and high between‐trial heterogeneity. After performing the review, significant heterogeneity was detected in the studies because 8 of the 12 studies were observational studies as opposed to randomized studies. Therefore, a random‐effects model (DerSimonian‐Laird method) was used throughout this analysis. The planned use of a fixed‐effects model was not employed. Outcomes were expressed as mean difference (MD) for continuous variables and odds ratio (OR) for dichotomous outcomes with 95% confidence intervals (CI).

Studies are presented separately as randomized and nonrandomized (observational) studies due to the heterogeneity and quality of the two types of studies.

A summary of the evidence was produced using the GRADE (Grading of Recommendations, Assessment, Development, and Evaluations) methodology.^22^

### 2.3 Results

**Figure 1:**
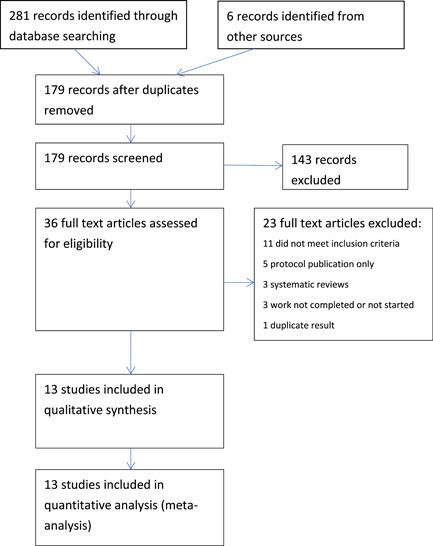
Preferred reporting items for systematic reviews and meta‐analyses flowchart of study selection.

After study selection, according to our predefined protocol, 36 full‐text manuscripts were reviewed and from these 13 studies, 3718 patients were suitable for inclusion in the review.^23^, ^24^, ^25^, ^26^, ^27^, ^28^, ^29^, ^30^, ^31^, ^32^, ^33^, ^34^, ^35^ (Figure 1) The studies excluded after full‐text review are presented in Supporting Information S1. Of the included studies, four were randomized trials (732 patients)^23^, ^25^, ^28^, ^32^ and nine were nonrandomized studies (2986 patients).^24^, ^26^, ^27^, ^29^, ^30^, ^31^, ^33^, ^34^, ^35^ Characteristics of individual studies are presented in Table 1. The first dose of IV iron was administered upon admission to the hospital in all but two studies. All studies compared the administration of intravenous iron to a control group or placebo. Three studies additionally compared the use of an erythropoiesis‐stimulating agent (erythropoietin, EPO) in conjunction with intravenous iron against the control group.^25^, ^30^, ^31^

**Table 1:** Characteristics of studies included in this systematic review.

	Design	*N*	Duration of follow‐up	Population	Intervention	Timing of intervention	Comparator	Reports duration of stay	Reports mortality	Reports quality of life	Reports transfusions	Reports discharge hemoglobin
Bernabeu‐Wittel 2016 (Spain)	RCT	203	60 days postdischarge	Age >65 years	1. FCM 1000 mg IV once	Administered before surgery (first 48 h)	Placebo (usual care)	Yes	Yes	Yes	Yes	Yes
	multicentre											
				Hb 90‐120 g/L	2. FCM 1000 mg IV once plus EPO 40000 units SC once							

Bielza 2021 (Spain)	RCT	253	12 months	Age >70 years	Iron sucrose 200 mg IV, three doses	Administered on Day 1, Day 3, Day 5	Placebo (saline)	Yes	Yes	Yes	Yes	Yes

Blanco Rubio 2013 (Spain)	Retrospective, cohort	120	In hospital stay	Age >65 years	Iron sucrose 200 mg IV, three doses	Administered on day of admission	Historical cohort	No	Yes	No	Yes	No

Clemmenson 2021 (Denmark)	Cohort	210	30‐day mortality	Acute hip fracture	Iron isomaltoside 20 mg/kg single dose ± blood transfusion	Administered on Day 3 post‐op if Hb < 104 g/L	Historical cohort who did not receive IV iron, but still had blood transfusion	Yes	Yes	No	No	No
				Hb <104 g/L on Day 3								
Cuence Espierrez 2004 (Spain)	Cohort	127	30‐day mortality	Age >65 years	Iron sucrose 100 mg IV once	Administered on Day 1, Day 2, Day 3	Historical cohort	Yes	Yes	No	Yes	No

Cuenca 2005 (Spain)	Cohort study, historical control group	77	30‐day mortality	Age >65 years	Iron sucrose 100 mg IV, up to three doses	First dose administered on admission, Dose 2 before surgery. A third dose was given between these timepoints, if the Hb < 120.	Historical cohort	Yes	Yes	No	Yes	No
				Hb <120 g/L								
Cuenca 2004 (Spain)	Cohort study, historical control group	157	30‐day mortality	Age >65 years	Iron sucrose 100 mg IV, up to three doses	First dose administered on admission, Dose 2 before surgery. A third dose was given between these timepoints, if the Hb < 120.	Historical cohort	Yes	Yes	No	Yes	No

Engel 2020 (USA)	Retrospective, case review	239	30‐day mortality and readmissions	Age >60 years	Iron sucrose 300 mg IV once at discretion of treating team if Hb <11 g/L	Administered when Hb < 110, either before or after surgery	Cohort during same time period who did not receive IV iron at discretion of treating team	Yes	Yes	No	Yes	No

				Proximal femoral fracture								
				Hb <110 g/L								
Garcia‐ Erce 2005 (Spain)	Prospective intervention (non randomized)	124	30‐day mortality	Age >65 years	Iron sucrose 100 mg IV 3 doses plus EPO 40000 units SC once	First dose administered on admission, then two further doses	Usual Care (No intervention)	Yes	Yes	No	Yes	No
				Hb <130 g/L								

Moppett 2019 (UK)	RCT	80	30‐day mortality	Age >70 years	Iron sucrose 200 mg IV 3 doses	Administered three doses on consecutive days starting within 24 h of admission	Control	Yes	Yes	No	Yes	No

Pareja Sierra 2019 (Spain)	Cohort, multiple arms, historical control	298	6 months	Age >75 years	1. Transfusion alone	Administered on day of admission and second dose 48 h later	Historical cohort	Yes^a^	Yes^b^	Yes^b^	Yes	No
					2. Iron sucrose 200 mg IV, 2 doses plus EPO 30000 units SC once							

					3. Transfusion plus Iron sucrose 200 mg IV 2 doses plus EPO 30000 units SC once							
Serrano‐Trenas 2011 (Spain)	RCT	196	30‐day mortality	Age >65 years	Iron sucrose 200 mg IV 3 doses	Administered on day of admission and second dose 48 h later	Placebo	Yes	Yes	No	Yes	No

Yoon 2019 (S. Korea)	Cohort	1634	3–5 years	Age >60 years	Iron sucrose 200 mg IV once plus lower transfusion trigger Hb < 80 or symptoms	Administered before surgery	Historical cohort with no iron and transfusion trigger Hb < 100	Yes	Yes	No	Yes	Yes


Abbreviations: EPO, erythopoetin; FCM, ferric carboxymaltose; Hb, hemoglobin; RCT, randomized controlled trial; SC, Subcutaneous.

^a^ This outcome is reported in the study group only, and not in the comparison group.

^b^ Data insufficient for analysis.

**Figure 2:**
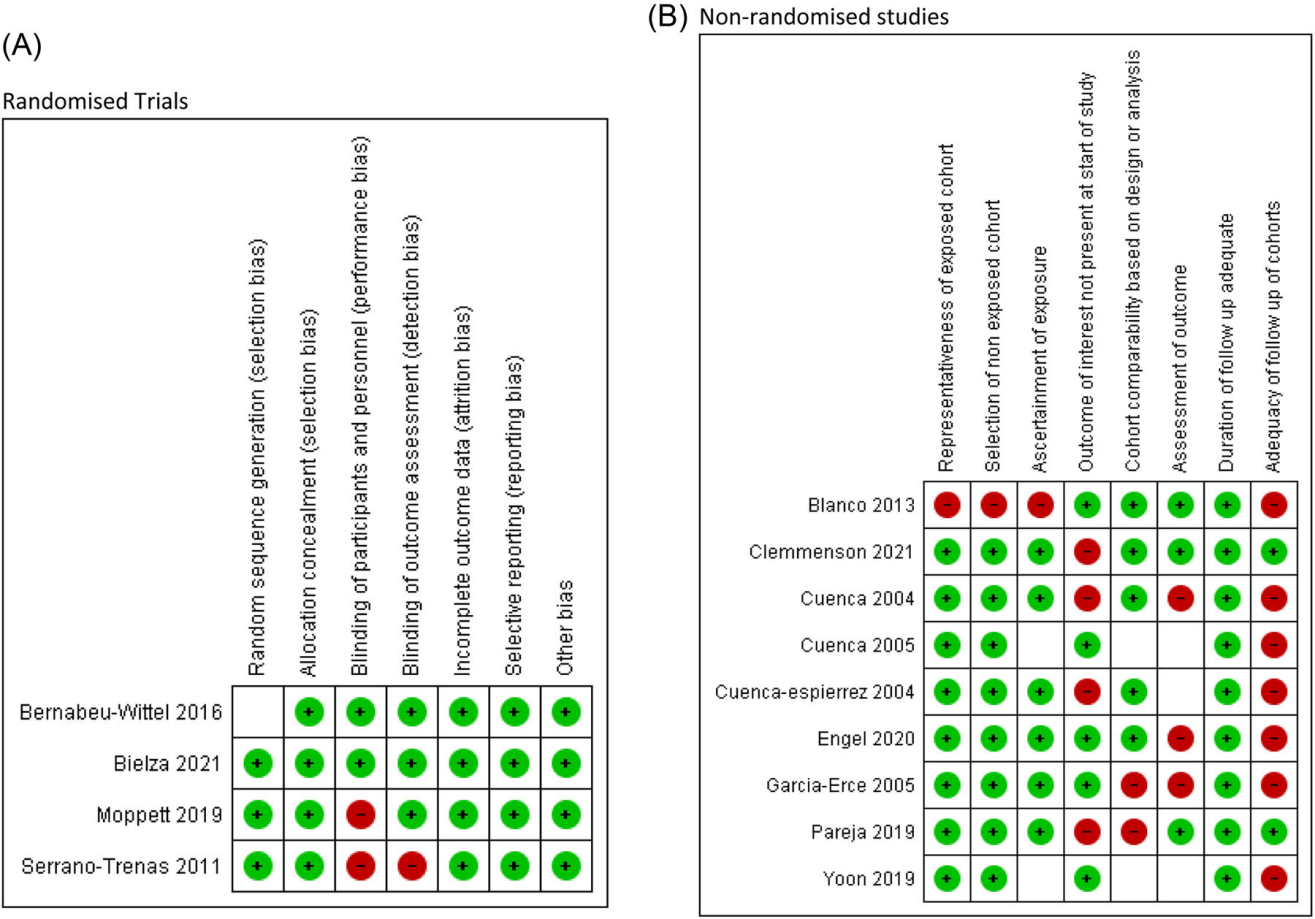
Risk of bias for (A) randomized trials (Cochrane risk of bias tool) and (B) nonrandomized studies (Newcastle Ottawa Scale).

The four randomized trials were assessed as having a low risk of bias; however, the nine nonrandomized cohort studies were of variable quality with a range of Newcastle‐Ottawa risk of bias scores between 4/8 and 6/8 (median score of 5/8) (Figure 2). Due to the risk of bias assessment, the RCTs and nonrandomized studies were analyzed separately and presented both separately and combined (the preponderance of nonrandomized studies returned by our search strategy was not known beforehand). No evidence of publication bias was detected (see Supporting Information S1). Nine of the 13 studies included were observational so we decided that using a random‐effects model for all analyses would be more appropriate due to the risk of heterogeneity.

### 2.4 Primary outcome

**Figure 3:**
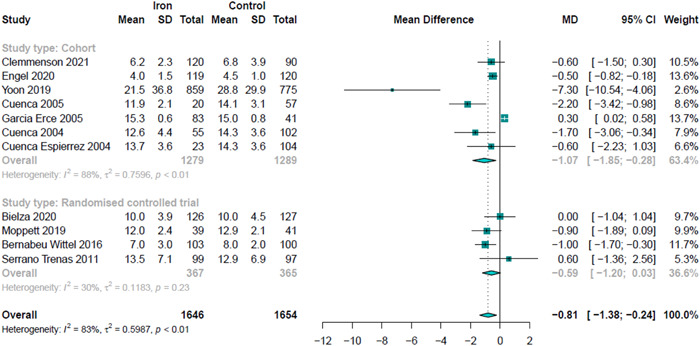
Sensitivity analysis for primary outcome, Length of Stay.

The primary outcome was reported in 3300 patients, 732 in RCTs, and 2568 in nonrandomized studies. In the RCTs, the MD in duration of the length of stay was 0.59 days less in those who received IV iron when compared to placebo or usual care: MD: −0.59, 95% CI: −1.20, 0.03; *I* ^2^ = 30%, *p* = 0.23 (Figure 3).

The sensitivity analysis for the nonrandomized studies returned a MD in length of stay of −1.07 days (95% CI: −1.85, −0.28; *I* ^2^ = 88%, *p* < 0.01). The duration of hospital stay data (mean and SD data) from one cohort study^33^ appears to represent an outlier study. A post‐hoc sensitivity analysis without the inclusion of this study is presented in Supporting Information: Figure S3.

### 2.5 Secondary outcomes

#### 2.5.1 Mortality

**Figure 4:**
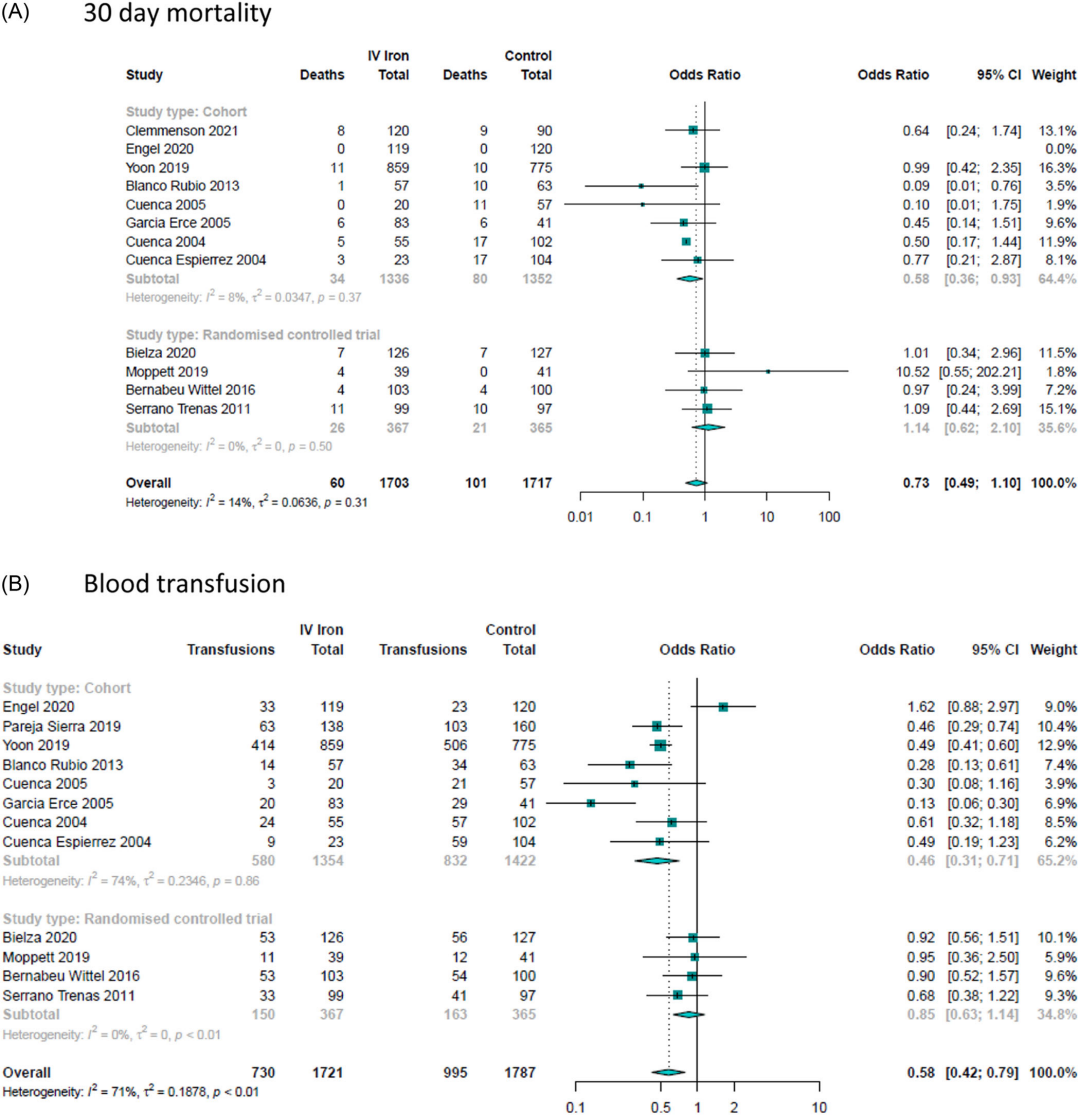
Forrest plot for secondary outcomes.

Four RCTs and eight nonrandomized studies, reported mortality at acute hospital discharge or 30 days,^23^, ^24^, ^25^, ^26^, ^27^, ^28^, ^29^, ^30^, ^32^, ^33^, ^34^, ^35^ including 732 and 2986 patients respectively. There was no difference between the groups in either the RCTs (OR: 1.14, 95% CI: 0.62, 2.1; *I* ^2^ = 0%, *p* = 0.50), nonrandomized studies (OR: 0.58, 95% CI: 0.36, 0.93; *I* ^2^ = 8%, *p* = 0.37) nor overall (OR: 0.73, 95% CI: 0.49, 1.10; *I* ^2^ = 14%, *p* = 0.31). Two studies including 1887 patients reported mortality at 90 days.^28^, ^33^ There was no difference between groups (Figure 4, Supporting Information: Figures S4 and S5).

### 2.6 Quality of life

Health‐related quality of life was measured in three studies (two RCTs).^25^, ^28^, ^31^ One measured health‐related quality of life using the Short Form 36 at baseline and 60 days after discharge from the hospital. Scores decreased from baseline to 60 days but they detected no difference in physical and mental component scores between the study groups.^25^ Another reported functional outcomes at 3 and 6 months after hip fracture.^31^ These were improved in the patients who received intravenous iron and erythropoietin; however, changes in the Barthel Index and Functional Ambulatory Category (FAC) scale between the groups did not reach statistical significance.^31^ The most recent study, from 2021, reported a change in Barthel Index during the initial hospital stay before discharge home as its primary outcome.^28^ There was no difference reported in the improvement in Barthel Index (over a median 10‐day hospital stay) with the intravenous iron intervention compared to placebo.^28^ The study was not powered to detect a difference in Barthel Index at 3 months.

### 2.7 Postoperative red cell transfusion

Twelve studies, including four RCTs (732 patients) and eight nonrandomized studies (2776 patients), reported red cell transfusion after surgery.^23^, ^25^, ^26^, ^27^, ^28^, ^29^, ^32^, ^33^, ^35^ In the RCTs, there was no difference in transfusions demonstrated: OR: 0.85, 95% CI: 0.63, 1.14; *I* ^2^ = 0%, *p* < 0.01 (Figure 4). In the nonrandomized studies, fewer transfusions were administered to patients who had received IV iron: OR, 0.46; 95% CI, 0.31, 0.71; *I* ^2^ = 74%, *p *< 0.86.

### 2.8 Discharge hemoglobin

Three studies, two RCTs^25^, ^35^ (456 participants) and one nonrandomized study^32^ (1634 participants) reported discharge hemoglobin. While a difference was demonstrated in discharge hemoglobin in the nonrandomized study, there was no difference in discharge hemoglobin between groups in the RCT (Supporting Information: Figure S6).

### 2.9 GRADE quality of evidence

Grade assessment of bias for each outcome was analyzed and presented in Table 2. Complete quality assessment for each outcome within RCTs rates the evidence as very low (quality of life, discharge hemoglobin), low (transfusion), and medium quality evidence (LOS and mortality) (Supporting Information: Figure S11).

**Table 2:** Risk of bias across primary and secondary outcomes for randomized studies (GRADE criteria).

	Sequence generation	Allocation concealment	Blinding of assessor	Blinding of participants	Blinding of personnel/clinicians	Incomplete outcome data	Selective outcome reporting	Other threats to validity	GRADE risk of bias assessment for this outcome	Overall GRADE risk of bias for this outcome
Primary outcome: Length of hospitalization 4 RCTs, *n* = 832										
Bernabeu Wittell	Low	Low	Low	Low	Low	Low	Low	Low	Low risk of BIAS	High quality evidence
Bielza	Low	Low	Low	Low	Low	Low	Low	Low	Low risk of BIAS	
Moppett	Low	Low	Low	Low^a^	Low	Low	Low	Low	Low risk of BIAS	
Serrano Trenas	Low	Low	Low	Low^a^	High^b^	Low	Low	Low	Low risk of BIAS	
Outcome: Mortality 4 RCTs, *n* = 832										
Bernabeu Wittell	Low	Low	Low	low	Low	Low	Low	Low	Low risk of BIAS	High quality evidence
Bielza	Low	Low	Low	Low	Low	Low	Low	Low	Low risk of BIAS	
Moppett	Low	Low	Low	Low^c^	Low	Low	Low	Low	Low risk of BIAS	
Serrano Trenas	Low	Low	Low	Low^c^	Low^d^	Low	Low	Low	Low risk of BIAS	
Outcome: Transfusion 4 RCTs, *n* = 832										
Bernabeu Wittell	Low	Low	Low	Low	Low	Low	Low	Low	Low risk of BIAS	High quality evidence
Bielza	Low	Low	Low	Low	Low	Low	Low	Low	Low risk of BIAS	
Moppett	Low	Low	Low	Low^e^	Low	Low	Low	Low	Low risk of BIAS	
Serrano Trenas	Low	Low	Low	Low^e^	High^f^	Low	Low	Low	Low risk of BIAS	

*Note*: GRADE table of quality assessment for primary and secondary outcomes: (a) LOS, (b) 30‐day mortality, (c) blood transfusion.

^a^ Participants are not blinded to treatment but this unblinding is unlikely to affect the LOS.

^b^ Clinicians are not blinded to treatment, this could affect LOS.

^c^ Participants are not blinded to treatment but this unblinding is unlikely to affect mortality.

^d^ Clinicians are not blinded to treatment but this unblinding is unlikely to affect mortality.

^e^ Participants are not blinded to treatment but this unblinding is unlikely to affect transfusion.

^f^ Clinicians are not blinded to treatment, this could affect blood transfusion.

## 3 DISCUSSION

The principal findings of this review were that perioperative treatment with IV iron in patients with hip fracture is not associated with a significant reduction in length of hospitalization, mortality, red cell transfusion, or discharge hemoglobin. We were unable to undertake a planned analysis on the effect of the intervention on health‐related quality of life as this outcome was infrequently reported and used a variety of metrics. Cost‐effectiveness and rates of infection were not examined in this study.

Another key finding from conducting this review was the paucity of work that has been published in this area: the published data comprised only four single‐center randomized trials and nine observational studies. Although the risk of bias in the RCTs was low, the overall GRADE quality of data for the RCTs was of medium to very low quality. The nonrandomized studies identified were heterogeneous and a comparison of these varied cohort studies, which exhibited heterogeneous methodology and outcomes provides only low‐quality evidence to complement the RCTs. There was marked variation between iron regimens and the use of adjuncts. The comparisons of changes in clinical practice with historic practice in an institution did not provide comparable controlled conditions to accurately evaluate new treatment regimens.

Our review presents up‐to‐date evidence in this field although similar reviews have previously been undertaken.^3^, ^36^, ^37^ This area of research has evolved since a narrative systematic review in 2010 reported a high incidence of perioperative anemia in a mixed orthopedic population; however, in this review, only two studies considered IV iron in hip fracture patients.^3^

Another meta‐analysis, in 2011, considered the effect of iron supplementation (oral or intravenous) versus no iron/placebo for treating anemia in a combined group of hip fracture and knee surgery patients.^36^ Meta‐analysis reported an increase in hemoglobin associated with oral iron treatment (*n* = 855). In contrast to our review, which included only hip fracture patients, this study included combined emergency and/or elective orthopedic patients. Moreover, five of the six studies analyzed oral iron supplementation and only one used intravenous iron. The most recent systematic review included all forms of iron supplementation in the elderly undergoing hip fracture surgery and included six RCTs (*n* = 1201) in the meta‐analysis.^37^ Four of these studies are included in our analysis;^25^, ^27^, ^29^, ^32^ however, two further studies were excluded in our review due to methodological concerns^38^ and the use of oral iron.^39^ In this analysis, length of stay and red cell transfusion were reduced in the intervention group and mortality was not affected. The authors also concluded that infection was not increased with iron treatment.

As the treatment of anemia with intravenous iron is increasingly adopted into clinical practice, it is important that this therapy is supported by high‐quality evidence. A recent large meta‐analysis has questioned the benefits of PBM programs in patients presenting for major surgery and has not demonstrated an overall benefit to mortality.^14^ Further studies might be better focused on the effect that this intervention has on recovery and patient‐reported outcomes rather than an absolute reduction in mortality. In another study, postoperative recovery after abdominal surgery was improved after IV iron despite there being no significant difference in the primary outcome of blood transfusion.^15^ Improvements in quality of life and other health‐related quality of life measures may be a more valuable endpoint in people with hip fractures.

The strengths of this review include adherence to a registered predefined protocol, PRISMA guidance, and Cochrane recommendations. However, the limitations of the available evidence have resulted in weaknesses in the analysis. We were unable to analyze data pertaining to health‐related quality of life, which was a prespecified outcome, due to inconsistency of reporting in the included studies. Searching returned a low number of randomized studies and a high number of nonrandomized cohort studies. We included analyses of separated and combined data to utilize as much patient data as possible, accepting that the nine cohort studies presented lower quality, heterogeneous evidence with a moderate risk of bias. Many of the cohort studies retrospectively compared changes in treatment plans (e.g., the introduction of intravenous iron to a clinical pathway) with groups of historical patients labeled as “usual care.”

Other variations in study characteristics import weakness to the analysis. The variation between intravenous iron regimens and the definition of anemia should be considered (Table 1). Eleven studies reported the use of intravenous iron sucrose: one randomized study utilized ferric carboxymaltose.^25^ Iron sucrose dose ranged from 100 to 300 mg, given either once, twice, or three times perioperatively. In contemporary clinical practice, a more usual dose of intravenous iron administered in the preoperative period to elective patients is a higher, weight‐based dose of either iron isomaltoside or ferric carboxymaltose in one or two infusions, respectively. Iron sucrose may be more representative of European clinical practice, as is the combination of intravenous iron with erythropoietin in three of the studies.^25^, ^30^, ^31^ The definition of anemia used was also variable (Table 1), and no studies confirmed iron deficiency. Last, one study^33^ with high weighting due to the number of patients included was a significant outlier within the primary outcome of the duration of hospital stay. Heterogeneity has resulted in all outcomes being downgraded for inconsistency during quality assessment using the GRADE classification.

In summary, the clinically relevant effect of IV iron in this population is unclear and medium‐ very low GRADE RCT evidence is published alongside a preponderance of cohort studies. Despite this, interest and use of intravenous iron in hip fracture patients is increasing. We therefore believe it is important to highlight the paucity of available evidence to allow clinicians to inform their clinical decisions. Better, large, high‐quality studies employing contemporary optimized drug regimens and measuring outcomes important to patients and clinicians are needed to answer the questions raised in this review.

## AUTHOR CONTRIBUTIONS

*Conceptualization*: Rhona C. F. Sinclair, Iain K. Moppett, and Michael A. Gillies.  *Methodology*: Rhona C. F. Sinclair, Iain K. Moppett, and Michael A. Gillies. *Formal analysis*: Rhona C. F. Sinclair, Miranda J. A. Bowman, Iain K. Moppett, and Michael A. Gillies. *Writing, original draft*: Rhona C. F. Sinclair. *Writing, review, and editing*: Rhona C. F. Sinclair, Iain K. Moppett, and Michael A. Gillies. All authors have read and approved the final version of the manuscript. Rhona C. F. Sinclair had full access to all of the data in this study and takes complete responsibility for the integrity of the data and the accuracy of the data analysis. The author, Rhona C. F. Sinclair, affirms that this manuscript is an honest, accurate, and transparent account of the study being reported; that no important aspects of the study have been omitted; and that any discrepancies from the study as planned have been explained.

## CONFLICT OF INTEREST

R. C. F. Sinclair has received an honorarium from Pharmacosmos UK. I. K. Moppett is Deputy Director of the Health Services Research Centre. I. K. Moppett is the colead of the Perioperative Specialist Interest Group of the Fragility Fracture Network and a member of the Quality Standards Group for NICE. I. K. Moppett has received research funding for studies into the perioperative management of hip fracture and his department has received consultancy funding from Astra Zeneca for work unrelated to hip fracture, blood transfusion, or intravenous iron. I. K. Moppett did not perform data extraction from the Moppett 2019 study. M. A. Gillies is a Chief Scientist's Office Scotland NHS Research Scheme Clinician. M. J. A. Bowman has no competing interests.

### TRANSPARENCY STATEMENT

We confirm that this manuscript represents an honest, accurate and transparent account of the study undertaken. Discrepencies from planned analysis have been explained within the methodology section.

## ETHICS STATEMENT

Ethical approval and patient consent were not required for systematic review. The review protocol was registered prospectively at Prospero. CRD42020171197. www.crd.york.ac.uk/PROSPERO.

## Supporting information

Supplementary information.

## Data Availability

The authors confirm that the data supporting the findings of this study are available within the article and its supplementary materials.
