# Supplementary material for: Perioperative intravenous iron to treat patients with fractured hip surgery: A systematic review and meta‐analysis
Source: Health Sci Rep. 2022 May 22;5(3):e633. doi: 10.1002/hsr2.633 (PMC9125168; doi:10.1002/hsr2.633)
Supplement: Supplementary file 1 — Supplementary information. [file HSR2-5-e633-s001.docx]

**Supplementary Files Table of Contents.**

1. Search strategy

2. Table of studies excluded after full text review

3. Sensitivity analysis for primary outcome, Length of stay, excluding outlier(Yoon)

4. Forrest plot for 90 day mortality

5. Forrest plot for 60 day mortality

6. Forrest plot for discharge haemoglobin

7. Funnel plot for primary outcome, Length of stay

8. Funnel plot for primary outcome, Length of stay, excluding outlier(Yoon)

9. Funnel plot for 30 day mortality

10. Funnel plot for red cell transfusion

11. GRADE summary table for assessment of RCT quality

## Supplementary File 1. Search strategy for Perioperative intravenous iron administration in patients with hip fracture: a systematic review.

The strategy was registered with Prospero in March 2020. The following search strategy is structured using a PICO model:

**EMBASE:**

1. (antianemic agent, or Iron, or Iron*, or intravenous iron, or IV iron, or iron compounds, or ferrous, or ferrous sulfate, or ferr*, or erythropoietin, or erythropoiesis stimulating agents, or EPO, or epo*, or B12, or folate). ti

2. (anaemia or anemia). ti

3. intravenous iron.ti

4. 1 OR 2 OR 3

5.( Hip surgery, or hip fracture, or femoral neck fracture, or proximal femur fracture, or femur fracture, or (hip adj3 surgery) or (femor* adj3 surgery) or (hip adj3 fracture) or (femor* adj3 fracture)). ti

6. 4 AND 5

Limits applied dates: 1.1.2000 – 1.3.2020

**MEDLINE**

1. (Iron, or Iron compounds, or ferrous*, or ferric*, or ferr*, or erythropoietin, or EPO, or epo*, or Vitamin B 12, or folic acid). ti

2. (anaemia or anemia). ti

3. intravenous iron.tw

4. 1 OR 2 OR 3

5.( femoral fractures, or hip fractures, or femoral neck fractures, or (hip adj3 surgery) or (femor* adj3 surgery) or (hip adj3 fracture) or (femor* adj3 fracture)). ti

6. 4 AND 5

Limits applied dates: 1.1.2000 – 1.3.2020

**CENTRAL AND DARE**

1. MESH descriptor Iron compounds

2. MESH descriptor haematinics

3. MESH descriptor anemia

4. MESH descriptor femoral fractures

5. MESH descriptor hip fractures

6. (#1 OR #2 OR #3)

7. (#4 OR #5)

8. (#6 AND #7)

Limits applied dates: 1.1.2000 – 1.3.2020

**CLINICAL TRIALS.GOV**

(hip fractures OR Hip injuries OR femoral fracture OR femoral neck fracture) AND (iron OR “intravenous iron” OR IV iron OR iron compounds OR ferrous OR ferrous sulphate OR erythropoietin OR EPO OR B12 OR folate)

Limits applied dates: 1.1.2000 – 1.3.2020

**ISRCTN**

1. ‘intravenous iron’

2. Refine results by ‘surgery’

Limits applied dates: 1.1.2000 – 1.3.2020

## Supplementary file 2. Table of Excluded Studies after full text review

| **Study** | **Reason for exclusion** |
| --- | --- |
| Bernabeu-Wittel 2012 | Protocol only |
| Bielza Galindo 2018 | Protocol only |
| Cochrane Central Trial registration ID 01972901 (2019)* | Protocol registration: study in progress |
| Garcia Erce 2015 | Letter to editor |
| Garcia Erce 2013 | Letter to editor |
| Garci Erce 2009 | Does not meet inclusion criteria |
| Izuel Rami 2008 | Does not meet inclusion criteria |
| Munoz 2004 | Duplicate publication of data |
| Clinical trials.gov registration NCT02972294 (2016)* | Protocol registration: study in progress |
| Pavesi 2013 | Does not meet inclusion criteria |
| Rowlands 2013 | Protocol only |
| Spahn 2010 | Systematic review |
| Yang 2011 | Meta-analysis |
| Saez lopez 2015 | Does not meet inclusion criteria |
| Xie 2015 | Does not meet inclusion criteria |
| Xu 2019 | Does not meet inclusion criteria |
| Goodnough 1996 | Does not meet inclusion criteria |
| Clinical Trials.gov registration NCT02428868 (2015) | This study was registered but not performed (email from CI) |
| Kim 2018 | Does not meet inclusion criteria |
| Clinical Trials.gov registration NCT01084122 (2010) | No report of this study found upon literature searching. Emails to study team no replies. |
| Munoz 2014 | Does not meet study criteria, and duplicate published data |
| Munoz 2012 | Dose finding study: still need to discuss: mixed group of elective and urgent pts. DW Mike |
| Chen 2020 | Systematic review |

*Indicates duplicate of the same study

S**upplementary Figure 3. Sensitivity analysis for primary outcome, LOS, with outlier study (Yoon) excluded.**

Mean difference (MD); standard deviation (SD); confidence interval (CI).


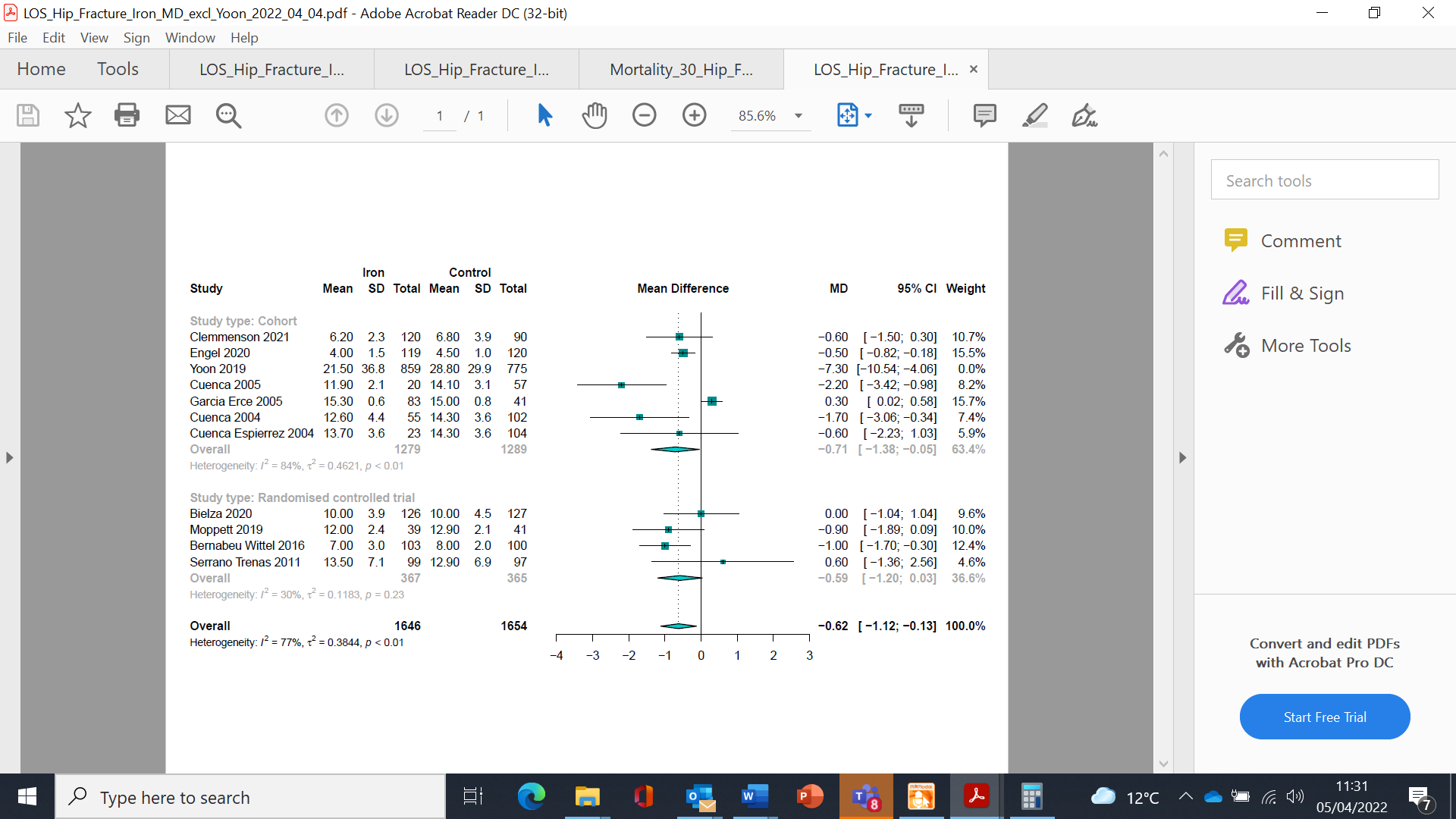


S**upplementary Figure 4. Forest plot for 90 day mortality.**


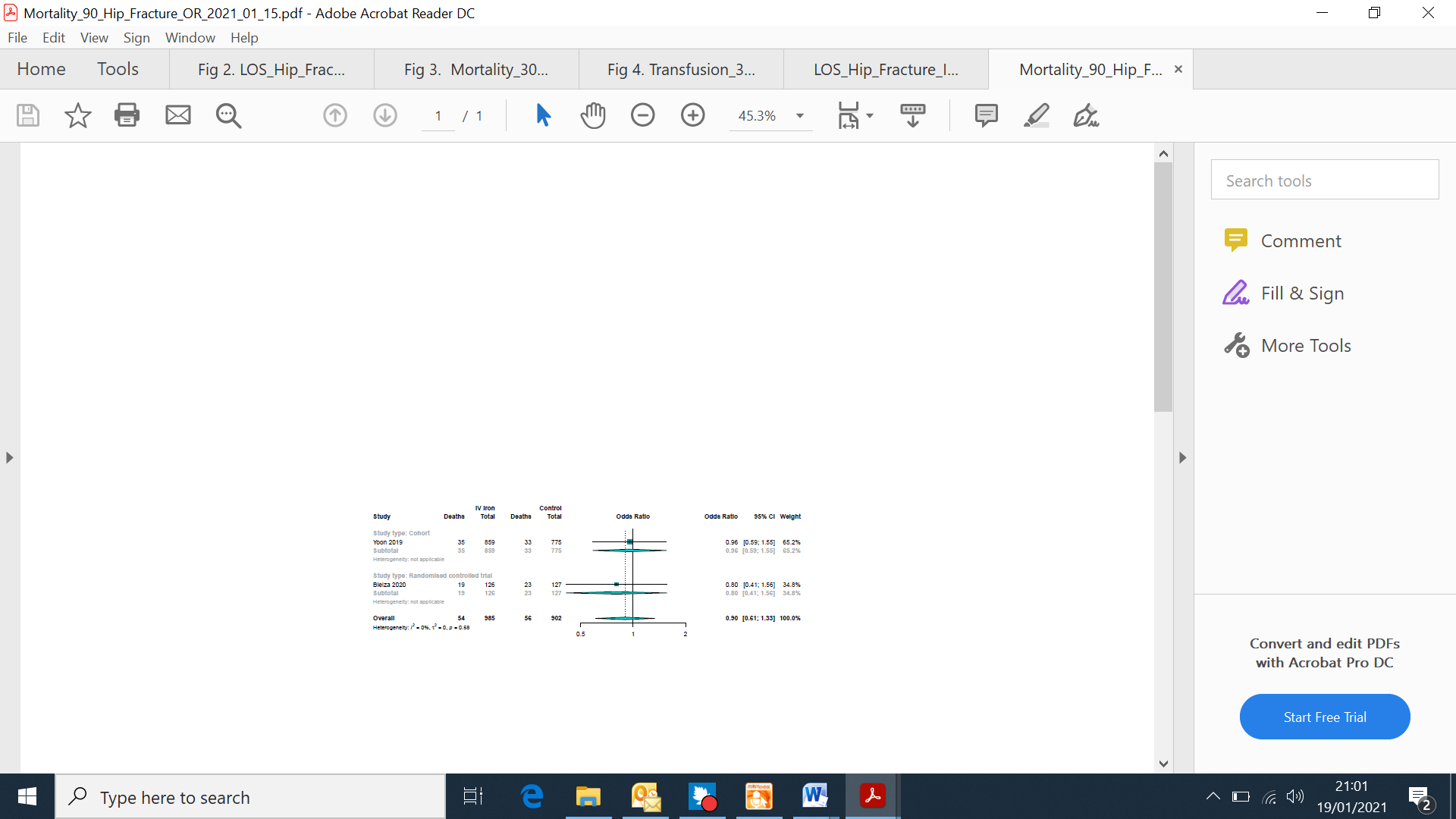


S**upplementary Figure 5. Forest plot for 60 day mortality.**


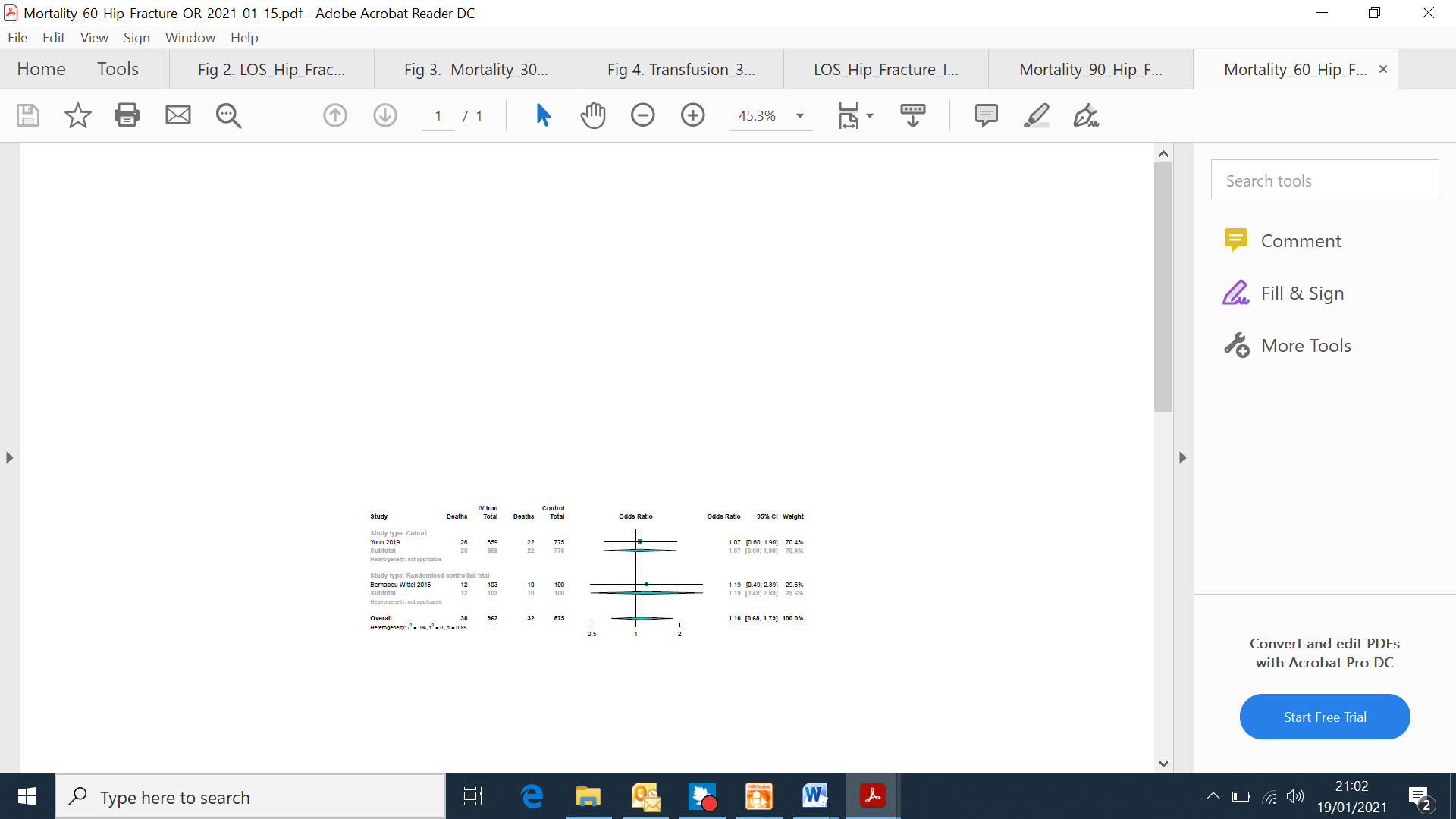


S**upplementary Figure 6. Forest plot for discharge haemoglobin.**

Intravenous (IV); standard deviation (SD); confidence interval (CI).


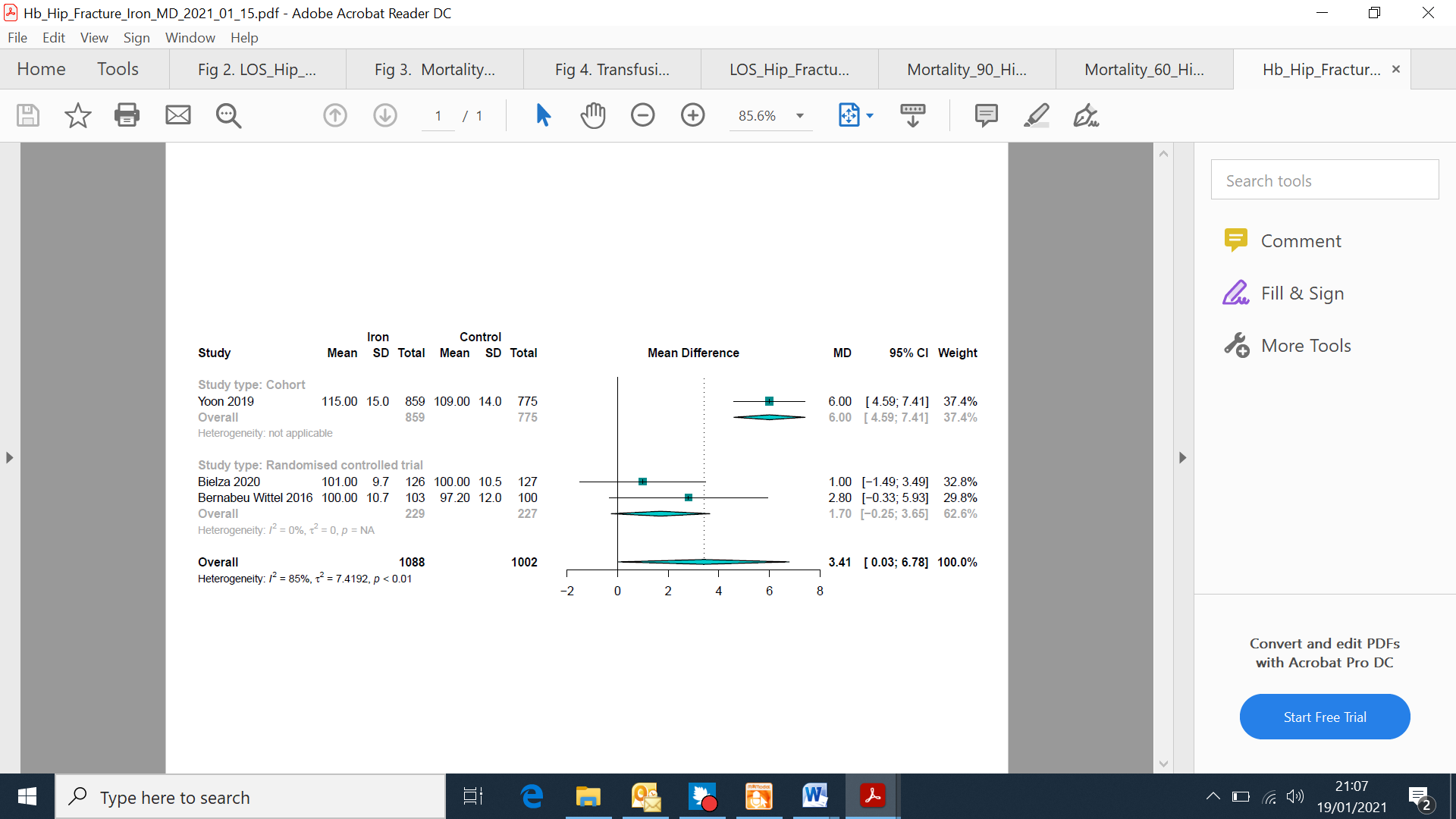


**Supplementary Figure 7. Funnel plot for primary outcome, Length of stay**


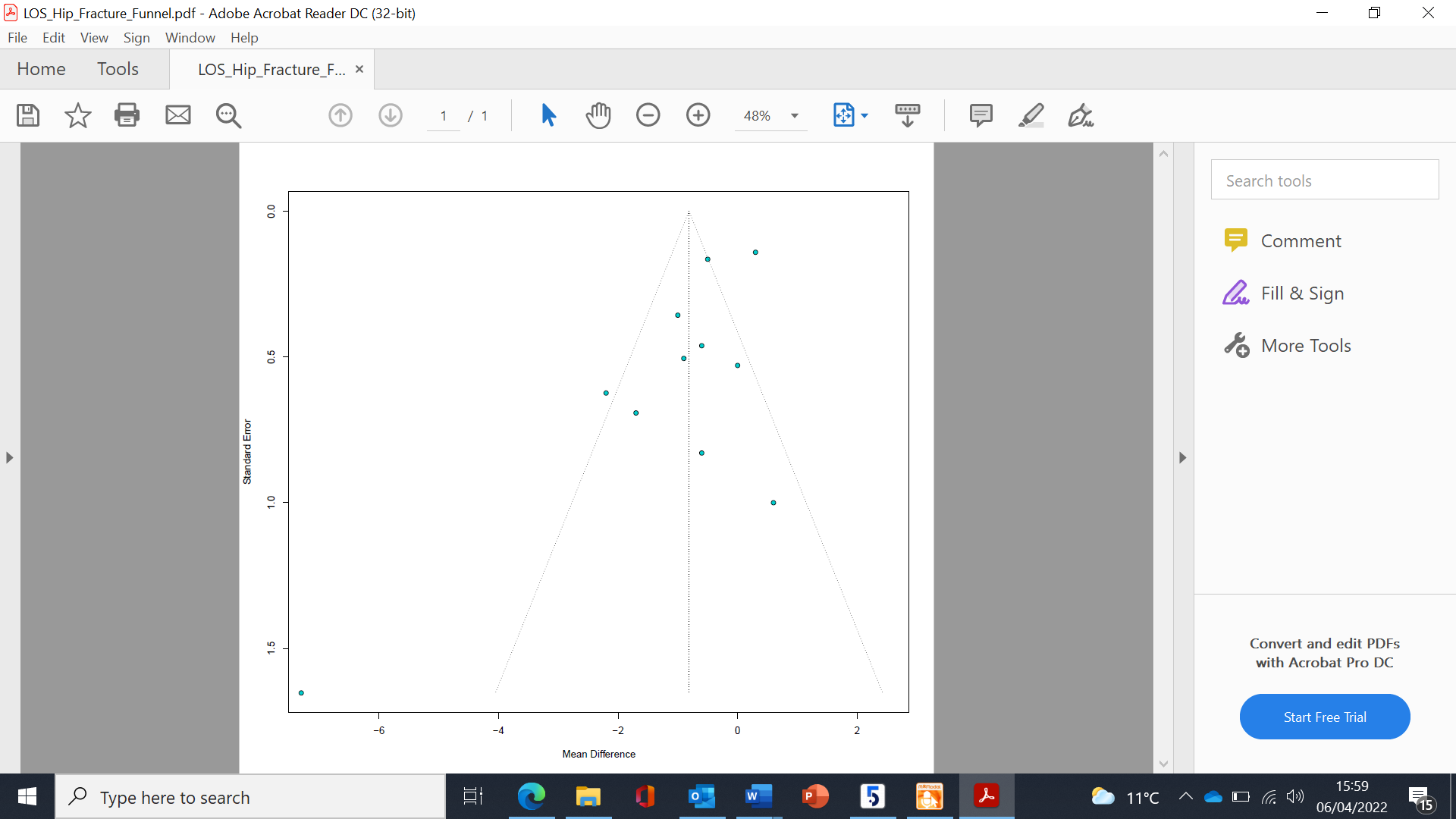


**Supplementary Figure 8. Funnel plot for primary outcome, Length of stay, excluding outlier (Yoon)**


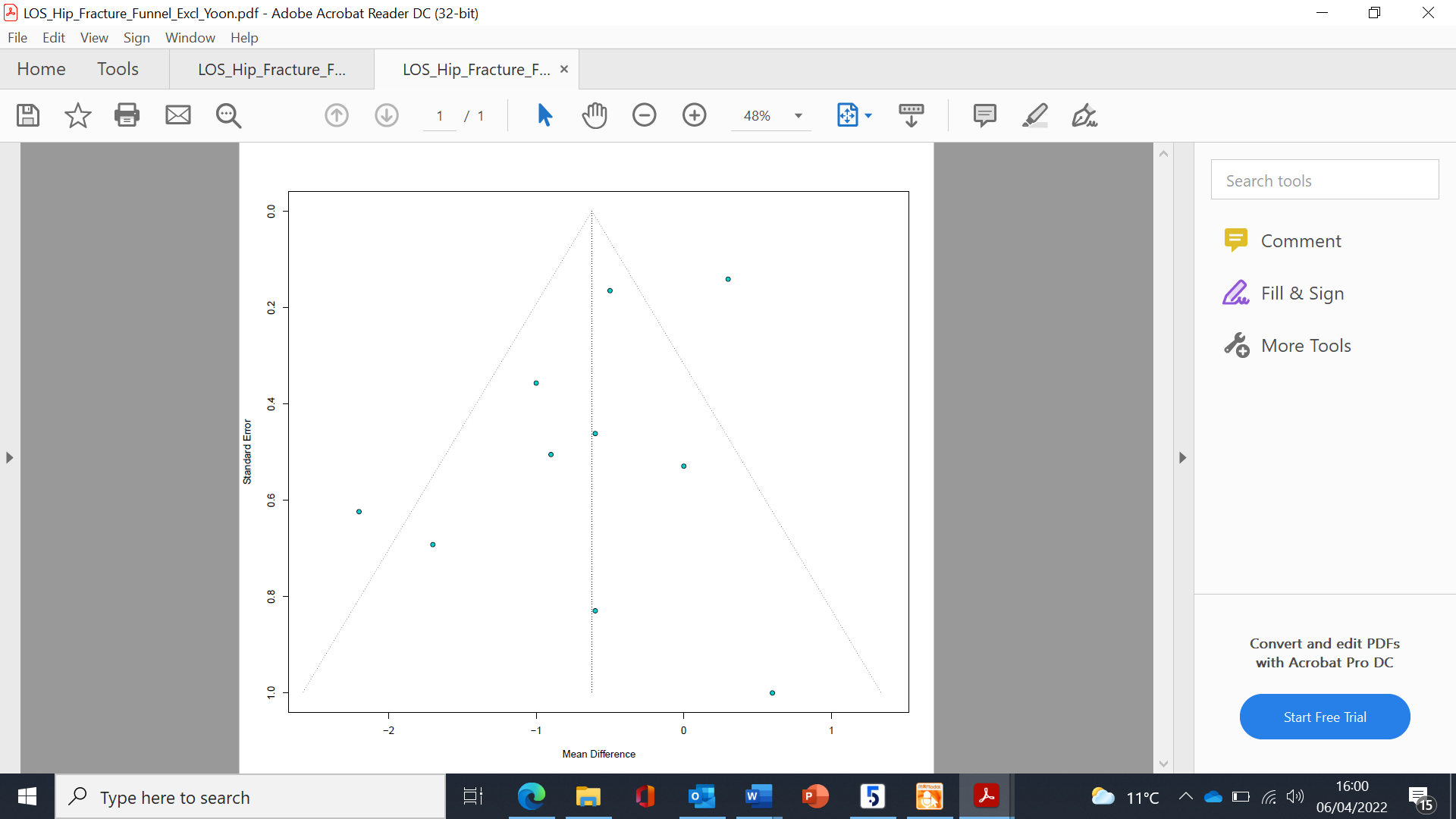


**Supplementary Figure 9. Funnel plot for 30 day mortality**


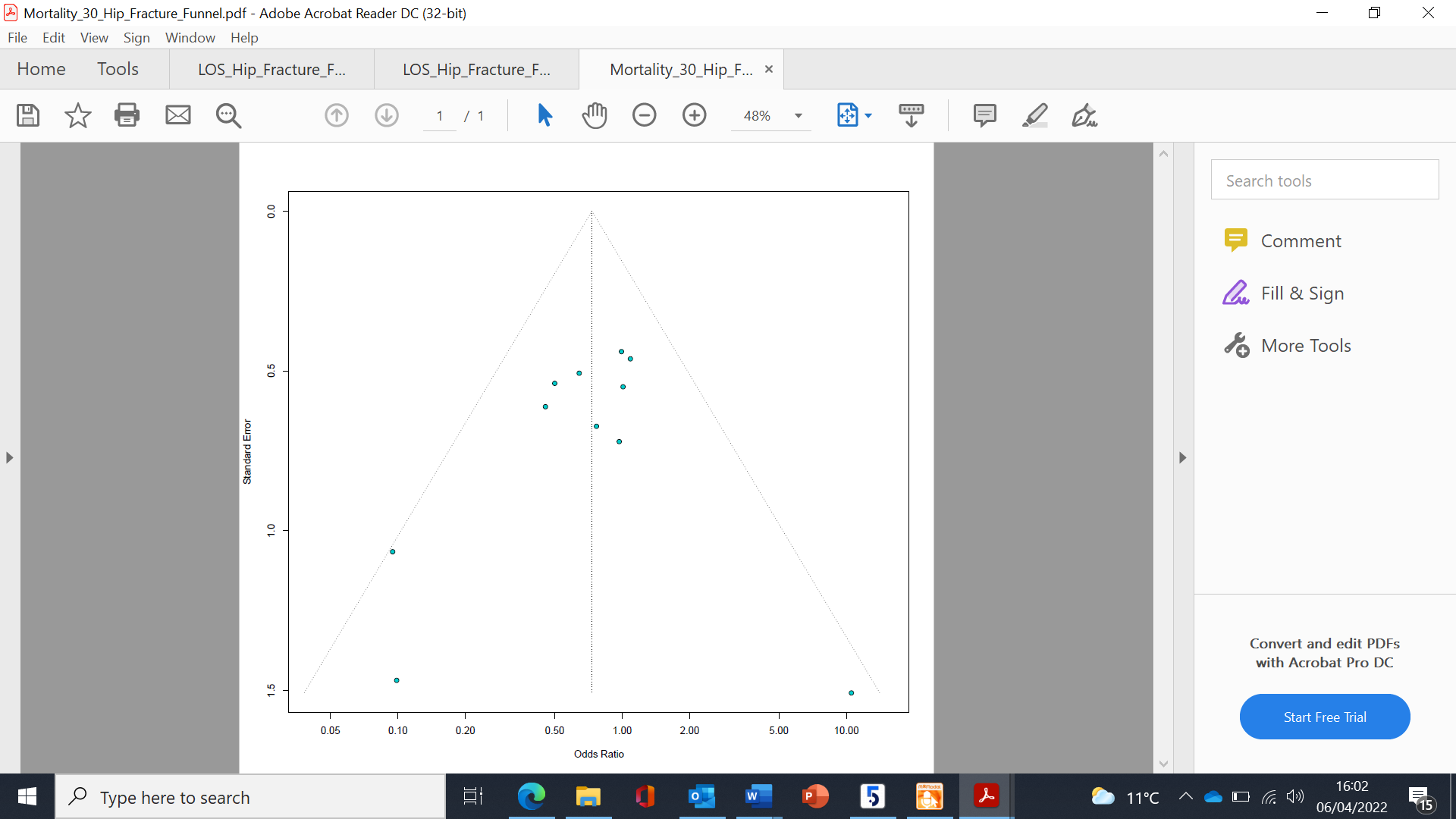


**Supplementary Figure 10. Funnel plot for red cell transfusion**


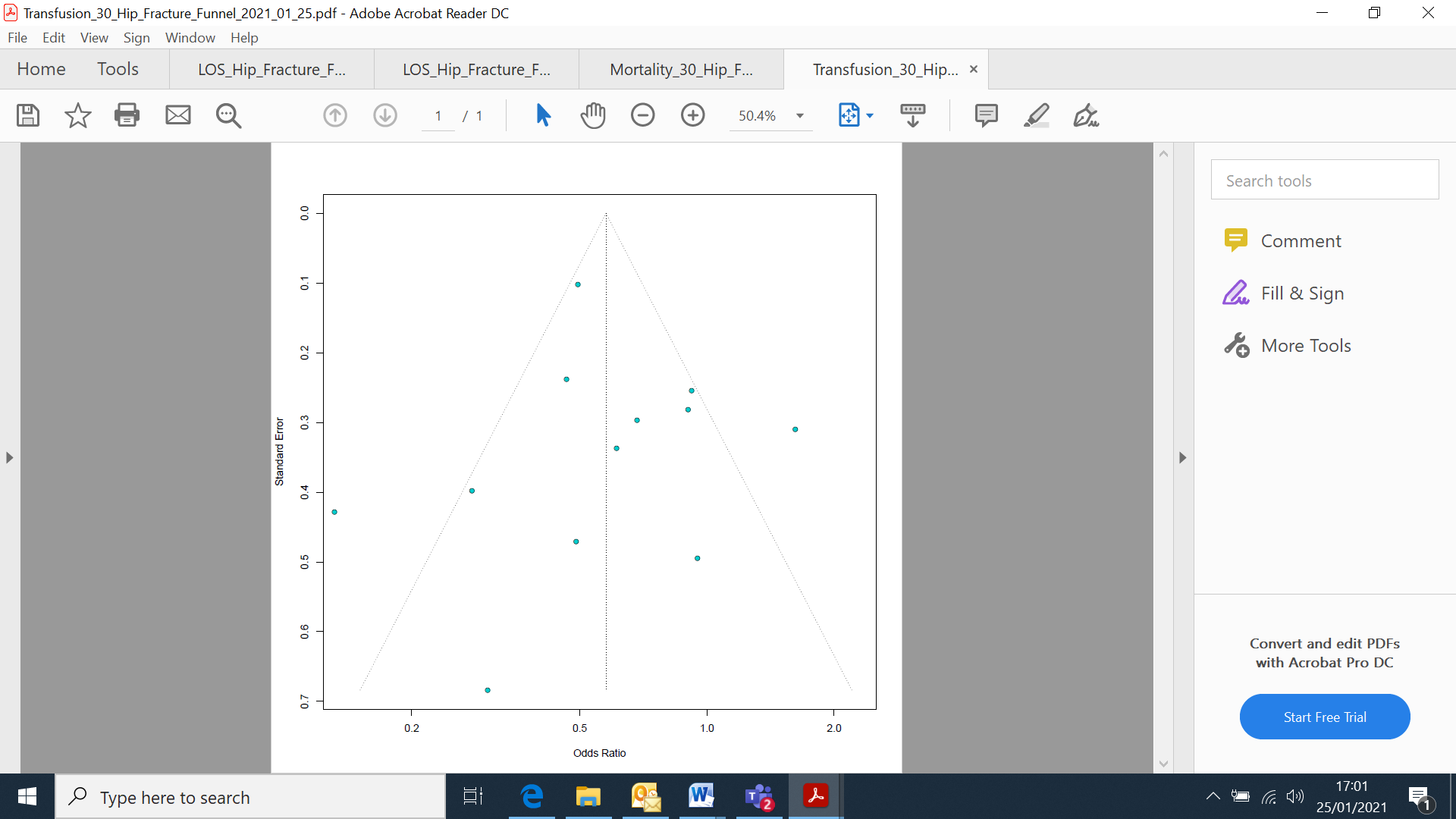


**Supplementary Table 11. GRADE assessment of quality of evidence**

GRADE table of complete quality assessment for RCTS

| Outcome | Risk of Bias | Imprecision | Inconsistency | Indirectness | Publication Bias | Overall quality assessment |
| --- | --- | --- | --- | --- | --- | --- |
| Length of Hospital Stay | Low risk of bias | Rate down for imprecision. CI crosses 0. | Rate down. Inconsistency relevant ( heterogeneity) | Not present | Not detected (funnel plot ok) | **Medium** |
| 30 day Mortality | Low risk of bias | Rate down for imprecision. OR is 1.0 with wide CI | Not relevant | Not present | Not detected (funnel plot ok) | **Medium** |
| Transfusion | Low risk of bias | Rate down for imprecision. CI crosses 0. | Rate down. Inconsistency relevant ( heterogeneity) | Not present | Not detected (funnel plot ok) | **Low** |
| Discharge Haemoglobin | Low risk of bias | Rate down for imprecision. CI crosses 0. | Rate down. Inconsistency relevant ( heterogeneity) | Not present | Strongly suspected (2 studies only) | **Very low** |
| Quality of Life | Low risk of bias | Unable to assess | Unable to assess | Not present | Strongly suspected (2 studies only) | **Very low** |
